# Targeting Epithelium Dysfunction and Impaired Nasal Biofilms to Treat Immunological, Functional, and Structural Abnormalities of Chronic Rhinosinusitis

**DOI:** 10.3390/ijms241512379

**Published:** 2023-08-03

**Authors:** Konstantinos Petalas, John Goudakos, George N. Konstantinou

**Affiliations:** 1 Department of Allergy, 251 General Air Force Hospital, 11525 Athens, Greece; kpetalas@hotmail.com; 2 Department of Otorhinolaryngology-Head and Neck Surgery, 424 General Military Training Hospital, 56429 Thessaloniki, Greece; jgoudakos@gmail.com; 3 Department of Allergy and Clinical Immunology, 424 General Military Training Hospital, Dorilaiou 10, Kalamaria, 55133 Thessaloniki, Greece

**Keywords:** polyps, eosinophilic, type 2 inflammation, biofilm, nitric oxide, microbiome, biomarkers, mucociliary clearance, cystic fibrosis, primary ciliary dyskinesia, immunodeficiencies, granulomatosis

## Abstract

Chronic rhinosinusitis (CRS) with (CRSwNP) or without (CRSsNP) nasal polyps is a prevalent and heterogeneous disorder existing as a spectrum of clinical conditions with complex underlying pathomechanisms. CRS comprises a broad syndrome characterized by multiple immunological features involving complex interactions between the genes, the microbiome, host- and microbiota-derived exosomes, the epithelial barrier, and environmental and micromilieu exposures. The main pathophysiological feature is an epithelial barrier disruption, accompanied by microbiome alterations and unpredictable and multifactorial immunologic overreactions. Extrinsic pathogens and irritants interact with multiple epithelial receptors, which show distinct expression patterns, activate numerous signaling pathways, and lead to diverse antipathogen responses. CRSsNP is mainly characterized by fibrosis and mild inflammation and is often associated with Th1 or Th17 immunological profiles. CRSwNP appears to be associated with moderate or severe type 2 (T2) or Th2 eosinophilic inflammation. The diagnosis is based on clinical, endoscopic, and imaging findings. Possible CRS biomarkers from the peripheral blood, nasal secretions, tissue biopsies, and nasally exhaled air are studied to subgroup different CRS endotypes. The primary goal of CRS management is to maintain clinical control by nasal douching with isotonic or hypertonic saline solutions, administration of nasal and systemic steroids, antibiotics, biologic agents, or, in persistent and more severe cases, appropriate surgical procedures.

## 1. Introduction

Chronic rhinosinusitis (CRS) is a prevalent and heterogeneous disorder characterized by a spectrum of clinical manifestations and distinct pathophysiology. CRS is associated with a high degree of morbidity and a significant quality of life impairment [1,2] with long-lasting financial burden implications on patients and health systems [3,4]. The prevalence of CRS ranges from 7% to 27% in European countries, with a point estimate of 11% [5], and 11.9% to 17.0% in the USA, with a point estimate of 12% [6]. Among CRS patients, more than a quarter express nasal polyps (NP). In the total population, the prevalence of CRS with NP (CRSwNP) is estimated between 2.1% and 4.4% in Europe [7,8], 4.2% in the USA [9], 2.5% in South Korea [10], and 1.1% in China [11]. These differences can probably be attributed to genetic particularities affecting not only the epidemiology of the disease but also the underlying pathophysiology [12].

Recent research and clinical evidence suggest phenotypic and endotypic heterogeneity among CRS patients. This narrative review summarizes CRS’s complex pathophysiology, relevant biomarkers, and state-of-the-art diagnostic approaches and therapeutic strategies, underscoring the need for in-depth understanding of this entity to offer maximum patient care and precision medicine implementation.

## 2. Comorbidities and Atopic Status

Aspirin sensitivity, allergic rhinitis, and asthma have been associated with CRS, especially with accompanying peripheral eosinophilia [13]. It is not clear whether there is an association between allergy and CRSwNP or CRS without NP (CRSsNP). However, evidence suggests a potential association between perennial allergens such as molds and house dust mites [14] and certain CRS subtypes, including allergic fungal rhinosinusitis (AFRS) and central compartment atopic disease (CCAD) [15]. CCAD is a CRS variant with isolated polypoid and edematous changes of the central structures of the visceral cranium, including the posterior–superior nasal septum, middle and superior turbinates, and paraclinical evidence of central opacification of the paranasal cavities with peripheral clearing strongly associated with allergy sensitization compared to those with diffuse polyposis [16,17,18].

The duration of allergen exposure may affect disease progression to CRSwNP or CRSsNP. Most studies have focused on patients with CRSwNP, mainly associated with type 2 inflammation. In contrast, CRSsNP has a much more complicated pathophysiology and could be related either to an eosinophilic inflammation or may involve eosinophil-independent inflammatory patterns, including IL-17, IL-21, IL-22, IL-26, and other cells such as innate lymphoid cells [19].

Furthermore, there are additional subtypes such as CRS associated with cystic fibrosis (CF), adenoids hypertrophy, primary ciliary dyskinesia (PCD), immunodeficiencies, autoimmune vasculitis and granulomatous diseases such as eosinophilic granulomatosis with polyangiitis, granulomatosis with polyangiitis, and sarcoidosis [1].

## 3. Pathophysiology

CRS is a broad syndrome characterized by multiple immunological features involving complex interactions between the genes, the microbiome, host- and microbiota-derived exosomes, the epithelial barrier, and environmental and micromilieu exposures.

### 3.1. Genetics-Epigenetics

A genetic basis for CRS has long been suspected. Patients with CRS are more likely to report a similar family history than those without CRS [20,21]. However, CRS does not express a clear pattern of heritability with a well-defined disease phenotype. It is considered a more complex process involving multiple genes, all having weak effects contributing to varying degrees of penetrance.

Allergic rhinitis and allergic asthma are disorders that may be associated with CRS. Both express inflammatory features similar to those seen in CRS, and have well-established genetic components [22,23].

Furthermore, CF is caused by a deficiency in epithelial chloride transport due to mutations in the CF transmembrane conductance regulator (CFTR) gene. Nearly all persons with two CFTR mutations and CF will develop CRS, while those with a single CFTR mutation have a three-fold increased risk of developing CRS [24].

Patients with PCD have frequent upper airway involvement, manifested as persistent rhinosinusitis with watery nasal discharge that begins in early infancy. Nasal polyposis in PCD is relatively uncommon, reported in less than 15% of children [25]. A mutation in at least one of the 31 genes encoding the different portions of the structural arm of the cilia has been associated with ciliary dysfunction [26].

TAS2R38 polymorphisms, encoding for the bitter taste phenylthiocarbamide receptor in the tongue and nasal epithelium, have been linked to significant differences in the ability to trigger sinonasal innate immunity and clear gram-negative bacteria. These polymorphisms have been associated with CRS and mainly CRSsNP [27]. Additional taste receptor gene variants, such as TAS2R19, may also play a role or predict a progression to CRS [28].

Persistent *Staphylococcus aureus* (*S. aureus*) infection has been associated with CRS and may contribute to NP formation [29]. *S. aureus* carriage has been associated with several genes loosely organized along reduced engulfment of bacteria, inflammatory response modulation, and barrier element genes [30].

Environmental interactions may also influence gene function and expression, causing epigenetic alterations that can affect the phenotypic expression of the disease. Parameters such as cigarette smoking and *S. aureus* colonization have both been associated with increased CRS severity and are implicated in epigenetic modification [31,32]. In a multicohort study, a novel single nucleotide polymorphism in the cadherin-related family member 3 gene (the receptor for rhinovirus C) was associated with adult CRS. This suggests that certain genome–microbiome interactions may predispose to CRS development [33].

### 3.2. Barrier Disruption—Role of the Epithelium

**Figure 1:**
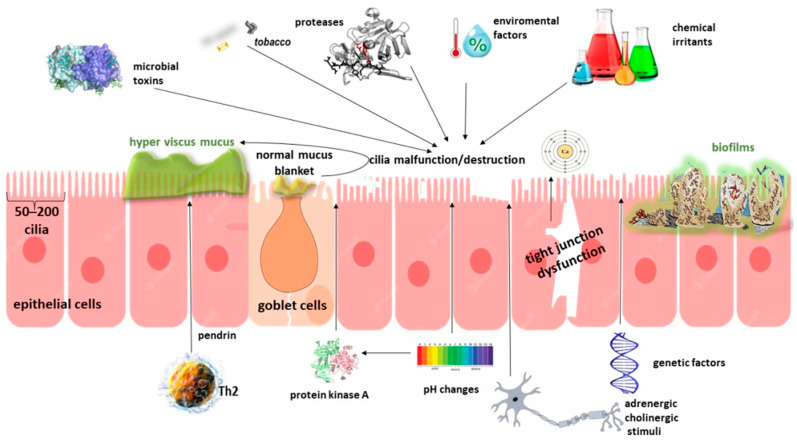
Defects in mucociliary clearance in chronic rhinosinusitis. Each epithelial cell is lined with 50–200 cilia. The overlying mucus blanket, produced by goblet cells interspersed among epithelial cells throughout the sinonasal mucosa and submucosal glands, possesses a dynamic gel-like composition in which its rheological properties tremendously influence mucociliary clearance. Ciliary activity accelerates in response to a variety of mechanical, chemical, hormonal, pH, and thermal stimuli. Furthermore, adrenergic and cholinergic stimulation have also been shown to stimulate ciliary motility. Th2 cytokine-mediated pendrin expression may increase mucus production. The activation of PKA coincides with an increase in CBF. An increase in intracellular pH produces an increase in CBF, whereas a decrease in pH produces a reduction in CBF, possibly due to PKA. Temperature has also been shown in many investigations to influence CBF, most likely through protein kinase C modulation. Lower temperature tends to slow CBF. Direct mechanical stimulation of the cilia increases CBF, which coincides with an increase in intracellular Ca^++^. CBF: ciliary beat frequency, PKA: protein kinase A.

In both CRSwNP and CRSsNP, the epithelium is known to be structurally and functionally abnormal, which may be crucial in the development and progression of CRS (Figure 1). Normally, the epithelial cells of the sinonasal mucosa, along with the overlying mucus layer, form a barrier to protect the host from exogenous factors and stimuli such as allergens, viruses, bacteria, and physical and chemical irritants and play an essential role in mucociliary clearance and innate defense. The epithelial cells are active by producing antimicrobial peptides, cytokines, and chemokines, activating intraepithelial and subepithelial cells, and recruiting these to respiratory tissues, thereby supporting a physical, chemical, and immunological barrier [34,35]. All extrinsic environmental factors can damage this epithelial barrier in CRS patients due to decreased expression of the tight junction proteins occludin-1, zonula occludens 1 [36], E-cadherin [37], and claudin-1 [38], resulting in a hyperreactive immune response [39]. Recent epidemiological evidence suggested an effect of ambient pollutants, such as particulate matter PM_10_ and PM_2.5_, on eosinophil recruitment, which could stimulate CRS establishment or be involved in already existing CRS progression [40].

The impaired epithelial barrier allows the microbiota (*Haemophilus influenzae*, *Streptococcus pneumoniae*, *S. aureus*, *Aspergillus fumigatus*, *Pseudomonas aeruginosa*) to translocate from the epithelium to the deeper layers beneath epithelial cells, to stimulate the immune system, contributing to pathogenetic inflammatory processes [41]. Microorganisms and common respiratory viruses disrupt the microtubule function of ciliated columnar cells and change the viscosity of surrounding mucus [42]. Consequently, the mucociliary clearance, which functions to clear the mucosal surface of particulates and irritants, is malfunctioning, hyper-viscous mucus is produced, and a vicious cycle of continuous abnormal over-exposure leads to immunological responses characterized by T2 inflammation or not [43]. A lot of cilia are either malfunction or completely destroyed [44].

Extrinsic pathogens, pollutants, and irritants interact with multiple epithelial receptors, such as pattern-recognition receptors (PRRs), which show distinct expression patterns, activate specific signaling pathways, and lead to diverse antipathogen responses. PRRs recognize microbial structures known as pathogen-associated molecular patterns (PAMPs) [45]. Toll-like receptors (TLRs) are a class of PRRs expressed on various immune cells, including B cells, specific T-cell types, macrophages, dendritic cells (DCs), fibroblasts, and epithelial cells. Expression of TLRs is modulated in response to pathogens, a variety of cytokines, and environmental stresses. TLRs regulate rapid antimicrobial peptides (AMPs) release by epithelial cells such as lysozyme, lactoferrin, antitrypsin, defensins, S100 proteins, and surfactants which play an important role in CRS [46]. Other antimicrobial peptides may have essential roles in the CRSwNP phenotype. The palate, lung, and nasal epithelial clone (PLUNC) family members, including SPLUNC-1, have antimicrobial and surfactant properties but are decreased in NP compared with healthy sinonasal tissue [47].

PRRs can promote the secretion of epithelial alarmins, including thymic stromal lymphopoietin (TSLP), IL-25, IL-33, and promote B-cell activation directly from the epithelial cells. These proinflammatory cytokines have been associated with CRS airway inflammatory responses [48]. Additionally, P-glycoprotein (P-gp) has been shown to be overexpressed in all CRS subtypes, especially on CRSwNP epithelium, where its stimulation directly promotes the secretion of these epithelial-derived cytokines [49].

Disturbance of the activity of proteases and their equilibrium with protease inhibitors seem capable of causing epithelial disruption and stimulation of cell-surface protease-activated receptors, specifically in Type-2-skewed endotypes of CRSwNP. These enzymes are excreted from aeroallergens such as house dust mites, pollens, fungi, or bacteria, including *S. aureus*, acting like “superantigens”, and *P. aeruginosa,* which, in turn, activates Th2 cells and group 2 innate lymphoid cells (ILC2s) to secrete T2 cytokines [49]. The environmental proteases have been, also shown to initiate epithelial-to-mesenchymal transition (ΕΜΤ) characterized by a loss of tight junctional proteins and transformation of basal epithelial cells to mesenchymal cells elaborating multiple extracellular matrix proteins such as desmin, fibronectin, tenascin, laminin, and collagens [50]. EMT occurs when adhesion disintegrates, resulting in the loss of its characteristic component, namely, E-cadherin [51]. Consequently, EMT markers, including TGF-β, elevated α-smooth muscle actin–positive activated myofibroblasts, increased fibronectin, and higher numbers of M2 macrophages are activated [52]. These markers, increased fibroblast activity, and extensive collagen deposition are associated with CRSwNP [53].

Trefoil factor 1 (TFF1), a highly conserved peptide expressed and secreted by epithelial cells in mucous membranes, has been shown to be involved in epithelial protection and repair processes. This factor is downregulated in established CRSwNP [54], while its upregulation can inhibit EMT and the underlying inflammation [55].

## 4. Inflammatory Endotypes in Chronic Rhinosinusitis

The inflammation in CRS is highly heterogeneous and can be subdivided into three main inflammatory endotypes. Firstly, the T1 endotype is associated mainly with the elevation of the T1 cytokines IFN-γ and IL-12, secreted from Th1 and ILC1s. Secondly, the T2 endotype is characterized by eosinophilia, ILC2s, and Th2 cells, and elevation of T2 cytokines (IL-4, IL-5, and IL-13) initiated by epithelial-derived IL33. Lastly, the T3 endotype is represented by neutrophilia, ILC3s, and Th17 cells, along with the elevation of T3 cytokines, including IL-17 and IL-22 [1,49]. Most studies, though, separated the inflammation into T2 and non-T2. However, Recent studies suggest that the molecular diversity of patients with CRS cannot reflect a simple differentiation only to two sub-endotypes (e.g., Th1 and Th2) [56,57].

### 4.1. Inflammation in CRSwNP

**Figure 2:**
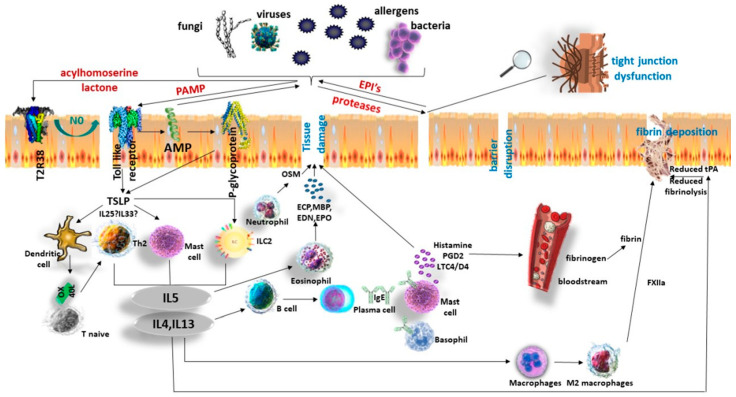
Mechanisms in chronic rhinosinusitis with nasal polyps. Exosome interacts with multiple epithelial receptors to induce both innate and adaptive immune responses. Protease exposure can lead to tight junction dysfunction and secretion of EPIs, which neutralize extrinsic proteases and stimulate type 2 inflammation. Similarly, PAMPs interact with TLRs, leading to AMP secretion. P-gp functions to clear the cytoplasm of environmental toxins while reinforcing epithelial cytokine release. Bacterial-derived acylhomoserine lactone interacts with T2R38 to induce NO release. As a result of these interactions, nasal epithelial cells induce TSLP (and perhaps IL-25 IL-33), which activates ILC2s, mast cells, upregulates OX40L expression on dendritic cells (DCs), and consequently pathogenic TH2 cells to produce type 2 cytokines. IL-5 recruits eosinophils which contribute to epithelium disruption. Neutrophils can also influence epithelial dysfunction. IL-4 and IL-13 activate epithelial cells, endothelial cells, macrophages, and B cells to induce barrier dysfunction and IgE-mediated reaction. Antigen/IgE/IgER complexes on mast cells and basophils induce degranulation and release prestored molecules, including histamine and enzymes that induce vascular leak and tissue damage. Plasma leak triggers fibrin deposition via cross-linking by FXIIIA released from M2 macrophages and reduction of tPA in epithelial cells. AMP: antimicrobial peptide, EPI: endogenous protease inhibitor, ILC2: group 2 innate lymphoid cell, NO: nitric oxide, PAMP, pathogen-associated molecular pattern, P-gp: P-glycoprotein, TLR: Toll-like receptor, tPA: tissue plasminogen activator TSLP: thymic stromal lymphopoietin.

CRSwNP is usually associated with moderate or severe Th2 eosinophilic inflammation. Up to 80% of such patients in Western countries have this endotype. On the contrary, up to 50% of East Asia patients with CRSwNP present without eosinophilic inflammation [58]. Th2 cells, mast cells (MCs), basophils, and ILC2s are accumulated in NP, contributing substantially to CRSwNP pathogenesis (Figure 2). T2 inflammation is mainly controlled by ILC2s and is not necessarily related to antigen presentation [56]. ILC2s are potent innate immune cells that can initiate T2 inflammation by producing IL-4, IL-5, and IL-13 [59]. Epithelial-derived innate cytokines IL-25, IL-33, and TSLP were identified as critical inducers of T2 cytokines in ILC2s. Viruses, protease-containing allergens, and the T2 cytokines IL-4 and IL-13 in epithelial cells intensify TSLP production [60]. The importance of IL-25 and IL-33 remains debatable [61,62,63]. ILC2s express a wide variety of receptors on their surface, some of which can induce the production of T2 cytokines when engaged with their respective ligands [64]. Activation of nuclear factor kappa-light chain-enhancer of activated B cells (NF-kB) and nuclear factor of activated T cells (NFAT) directly induces T2 cytokine production in ILC2s. The activation of signal transducer and activator of transcription (STAT)5 and STAT6 can potently enhance the induction of T2 cytokines in ILC2s when NF-kB or NFAT activators are present [65].

TSLP activates the STAT5 transcription factor and has been found to be significantly elevated in NP compared with the control sinus tissues [66].

The tumor necrosis factor (TNF) superfamily is a large group of cytokines with diverse regulatory functions during an immune response through the activation of NF-kB. Among them, only the receptor activator of NF-kB ligand (RANK-L) expressed in T_H_2 and ILC2 seems to be elevated in CRSwNP [67].

Lipid mediators, including prostaglandin D2 (PGD2) and cysteinyl leukotrienes (LTC4, LTD4, and LTE4), activate NFAT and are elevated in NP compared with healthy sinonasal tissue [68].

L-2 and IL-7, which signal through STAT5, are not potent inducers of T2 cytokines in ILC2s, but they can synergistically enhance the production of T2 cytokines generated through the activation of NF-kB or NFAT [64].

Numerous studies identified the elevation of T_H_2 cells in T2 NPs [69,70,71]. TSLP participates in Dendritic Cells (DCs)-mediated T_H_2 differentiation via the induction of OX40L on DCs. OX40L1 DCs are elevated in T2 NPs [71]. T_H_2 cells in NPs express receptor activators of nuclear factor kappa B ligand, which activates ILC2s to produce T2 cytokines [67]. ILC2 numbers significantly correlate with T_H_2 cells in sinus mucosa patients with CRSwNP [72]. Th2 cell subsets in CRSwNP control antigen-dependent T2 inflammation and are somewhat heterogeneous regarding their cytokine secretion and transcription factor profiles. In particular T_H_2 cells express GATA3, IL-17RB, ST2, HPGDS, and T2 cytokines [70]. Recently T_H_2 subpopulation expressed CD109 but lacked CRTH2 in NPs was shown by releasing not only T2 cytokines but also immunosuppressive IL-10 by having a transcription factor FOXP3 and behaving like Tr1 regulatory cells [73].

The elevated production of local polyclonal IgE plays a pathogenic role in CRSwNP via the activation of MCs and basophils [74] and is associated with eosinophilic inflammation and NP recurrence [57]. The presence of specific IgE against enterotoxins from *S. aureus* has been associated with nasal polyps exhibiting intense eosinophilic inflammation with very high IgE concentrations and concomitant asthma [57,75]. MCs and basophils are activated by cross-linking FcεRI via IgE-antigen/allergen to release mediators such as histamine, proteases, and T2 cytokines and synthesize lipid mediators CysLTs and PGD2. Different subsets of MCs have been characterized based on protease content: mast cell-tryptase (MCT), mast cell-tryptase/chymase (MCTC), intermediate MC between MCT and MCTC and proliferative MC (Ki671). MCTC and intermediate MCs play proinflammatory roles in T2 NPs [76]. However, local hyperimmunoglobulinemia is also present in non-atopic (as defined by the allergy nomenclature [77]) patients, meaning that elevated specific IgE levels result from other pathways, such as (i) ILCs and epithelial-derived cytokines [1], (ii) entopy, namely, the localized allergic inflammation that cannot be systemically measured [78], or (iii) autoallergy, against endogenous autoallergens in models similar to atopy eczema [79] or chronic spontaneous urticaria [80].

Macrophages are shifted to the M2 type, which is found elevated in T2 NPs and produces FXIII-A contributing to excessive fibrin deposition in the submucosa of NP [81,82,83].

Eosinophils are considered to be a hallmark of nasal polyps. The migration of eosinophils into the extravascular compartment is promoted by chemotactic factor receptors, such as CCR3, CRTh2, and CysLT1 [84]. Recruitment of eosinophils in CRSwNP is controlled by the local elevation of cell surface adhesion molecules, including P-selectin, β1 integrins, such as VLA-4, and β2 integrins, interacting with their respective counter-ligands expressed on inflamed endothelium of the sinus mucosa such as P-selectin ligand [CD162], VCAM-1, and ICAM-1 induced by IL-4 and IL-13 [56,84]. The prolonged survival of eosinophils in nasal tissue is supported by IL-5 and GM-CSF, protecting the eosinophils from apoptosis [56,84]. Eosinophils are locally activated in the nasal polyp tissue and express an increased cluster of differentiation (CD)69 [85]. Once activated, eosinophils secrete a broad range of substances, including eosinophil cationic protein (ECP), eosinophil-derived neurotoxin (EDN), eosinophil peroxidase (EPX), major basic protein (MBP), secretory phospholipase A2, galectin-10, Charcot-Leyden crystals, (CLC’s), enzymes, cytokines, growth factors, and chemokines, all contributing to tissue damage and promoting the inflammation [86]. Eosinophils can cause tissue damage by forming eosinophil extracellular traps composed of DNA, granule proteins, and CLC’s. Extracellular traps can increase the mucus viscosity [87] and lead to the entrap *S. aureus* and other bacteria [88]. CLCs are more than a degradation product of eosinophils because they affect the epithelial barrier and sustain a neutrophilic inflammation in CRSwNP [89].

Epithelial barrier-disrupting cytokine Oncostatin M (OSM) was detected in neutrophils in NP tissue and was elevated in T2 NPs [86].

### 4.2. Inflammation in CRSsNP

The molecular mechanisms in CRSsNP are poorly understood compared with those of CRSwNP. CRSsNP is mainly characterized by fibrosis and mild inflammation and is often associated with Th1 or Th17 inflammation. It may exhibit multiple inflammatory endotypes that show race or ethnic discrepancies. The inflammatory environment in CRSsNP has been classified into T1, T2, or T3 endotypes. The T2 endotype has become most common in Europe and USA, and 30% to 55% of patients with CRSsNP present the T2 endotype [90,91]. On the contrary, CRSsNP in patients from China has been found to be T1 predominant [91], as opposed to a mixed T1 and T3 or a T3-dominant inflammatory pattern found in Korean patients [92]. Both T1- and T3-related inflammatory endotypes of CRSsNP have been found to be controlled by distinct genes suggesting alternative underlying pathomechanisms that may explain the observed abnormal micromilieu in the nasal mucosa of CRSsNP patients.

In patients with CRSsNP exhibiting a T1-predominant biomarker profile, a group of 126 different genes was found upregulated, most of which were highly associated with the regulation of cells producing IFN-γ and IFN-γ signaling [93], including IFNG gene and genes associated with IFN-γ productions [94,95], MHC-related molecules, T cell, NK cell- and CD8+ T cell-associated genes, and molecules implicated in acute inflammation and host defense [96,97]. T1-mediated immunity protects against intracellular bacteria and viruses, and IFN-γ is a key immune effector cytokine produced from activated CD4+ T_H_1 cells as well as CD8+ cytotoxic T cells, NK cells, and ILC1 [98].

When examining the nasal mucosa of patients with a T3-related CRSsNP endotype, 545 genes were found upregulated [93], including genes involved in the production of proinflammatory and acute inflammatory cytokines, MHC class II molecules, complement proteins, and host defense proteins, and the accumulation, activation, and degranulation of neutrophils [96,97,99], B cells, T cells, and macrophages [93,100]. The observed upregulation of IL-17A- and IL-17F-associated genes could suggest the Th17 cells as the primary contributors of T3-prominent CRSsNP [93]. T3-mediated immunity provides host defense against extracellular microbes such as bacteria and fungi. IL-17A and IL-17F are key effector cytokines produced mainly from Th17 cells and ILC3 [98].

Lastly, the contribution of biofilm formation seems to be crucial for CRSsNP pathogenesis. Many microorganisms in the sinonasal tract contribute to a biofilm formation, comprised of a community of bacteria or fungi surrounded by a protective extracellular matrix that is more resistant to antibiotics and host defenses [101]. The presence of biofilms in the sinonasal tract is connected to recalcitrant CRS and worse outcomes after surgery [102,103].

## 5. Diagnosis of CRS

CRS in adults is defined as the inflammation of the nose and paranasal sinuses, persisting for more than 12 weeks and characterized by two or more symptoms, one of which should be either *nasal blockage/obstruction/congestion* or *nasal discharge* (*anterior/posterior nasal drip*), and additional symptoms could be facial pain/pressure or reduction or loss of smell and either.

1. *endoscopic signs* of: nasal polyps (NP), and/or mucopurulent discharge primarily from middle meatus and/or oedema/mucosal obstruction primarily in middle meatus, and/or
2. *CT-scan changes*: mucosal changes within the ostiomeatal complex and/or sinuses

In children, the only difference is that the clinical sign accepted is cough instead of loss of smell [1].

Additional symptoms such as oropharyngeal discomfort, otalgia, halitosis, dental pain, cough, malaise, headache, and fatigue may exist [104]. The diagnosis must be confirmed by objective findings on nasal endoscopy or computed tomography (CT) to improve the diagnostic accuracy [49].

### 5.1. CT-Scan Findings

CT imaging of paranasal sinuses without intravenous contrast is the radiologic modality of choice to establish the diagnosis of CRS. Conventional sinus radiographs are inaccurate in most patients [105]. Magnetic resonance imaging (MRI) is not considered the first-line study for routine sinus imaging because of the lack of bone detail and length of imaging time [106]. Furthermore, inspissated secretions may appear dark on T2 sequences, resembling air [107]. CT scanning provides excellent delineation of the complex ethmoidal anatomy, ostiomeatal unit, and anatomic variations, including the presence of sphenoethmoidal air cells, which increase the risk of injury to the optic nerves or carotid arteries. CT imaging can also be imported into computer navigation systems (electromagnetic and optical guidance) for image-based guidance surgery during endoscopic sinus surgery. MRI may be complementary in aggressive infections, invasive fungal sinusitis, or sinonasal masses [106].

The most commonly used staging system to quantify the changes observed on CT is Lund–Mackay (LM), which has been validated in several studies [108,109]. LM is based on the degree of opacification for the maxillary, anterior and posterior ethmoids, frontal and sphenoid sinuses (0—none; 1—partial; 2—complete), and ostiomeatal complex (0 or 2), giving a maximum score of 24 or 12/side [110]. CRS studies comparing symptoms with CT and endoscopic findings have shown a good correlation between CT and endoscopy but generally not between symptoms and CT. More than 40% of patients who fulfill the symptom-based diagnosis of CRS may have normal CT and endoscopy results [111].

The importance of CT imaging is obvious in phenotyping CRS as localized (unilateral) or diffuse (bilateral) [1]. The pattern of inflammation may also be important. The hallmark of inhalant/IgE-driven CRS is a central thickening of the turbinates and septum with near-normal peripheral sinus mucosa. This was initially described by Lund et al. as the “black halo” sign [112]. The degree of osteitis in CRS defined via CT imaging may have a multitude of associations with the clinical presentation of CRS and especially the recalcitrant phenotype [113].

### 5.2. Biomarkers

Possible CRS biomarkers could be originated in peripheral blood, nasal secretions, tissue biopsies, and nasally exhaled breath. The current definition of eosinophilic CRS is based on greater than 10 eosinophils per high-powered field (HPF) in mucosal tissue specimens collected from the ethmoid cavity, which has been generally associated with poorer outcomes and overall prognosis [114,115].

Blood eosinophilia is not definitively correlated with that in tissue [116]. Ho J et al. reported that more than 240/μL peripheral eosinophils could be a surrogate marker for tissue eosinophilia of more than 10/HPF [117]. A blood eosinophil count of more than 450/μL has been associated with the need for long-term systemic therapy following endoscopic sinus surgery in eosinophilic CRS cases, with a high negative predictive value (98.7%) [118].

Total serum IgE, positive skin prick testing, or serum-specific IgE have shown no significant association with CRS [116,119]. In contrast, comorbid allergy, elevated tissue IL-5, or IgE levels (including specific IgE against *S. aureus* enterotoxins) were significant predictors of the need for revision surgery [120].

Cytokine profiles, including IL-4, IL-5, IL-13, IL-25, IL-33, TSLP (in CRSwNP) and TGF-β, type I interferons, IL-6, IL-8, or IL-17 (in CRSsNP) in nasal tissue are perhaps the most investigated and promising biomarkers for phenotyping CRS and targeting therapeutically [121]. Biologic agents, including omalizumab (anti-IgE), mepolizumab (anti-IL-5), and dupilumab (anti-IL4/13), have been approved for CRSwNP treatment, confirming the pathogenetic role of the targeted immunoglobulin/cytokines.

Periostin is an extracellular protein that is secreted in response to IL-4 and IL-13. It plays a role in airway subepithelial fibrosis through interactions with integrin molecules involved in tissue remodeling [122]. Periostin is elevated in CRSwNP patients, especially when the disease is active [121]. Consequently, there is potential for periostin itself to be used not only as an activity biomarker but also as a viable target for reducing inflammation.

P-glycoprotein is related to T_H_2 inflammation through the promotion of cytokine secretion. P-glycoprotein levels are elevated in all CRS subtypes and can be in nasal fluids [123].

Eosinophilic granule proteins, including MBP, ECP, EDN, and EPO are responsible for airway inflammation, tissue damage, and remodeling and are markers of eosinophilic activation [124]. ECP has been shown to be increased in the serum of CRSwNP patients and correlated with both raised blood eosinophils and increased eosinophil concentration on nasal smears [125,126]. Serum EDN levels are significantly elevated in eosinophilic CRS compared with non-eosinophilic CRS or controls and correlate with polyp score (the nasal polyp score scale ranges from 0 (no polyp) to 4 (large polyps) for each nostril) and peripheral eosinophilia [126]. On the other hand, no association has been identified with regard to serum EPO and eosinophilic CRS. The patients with CRSwNP have decrements in exhaled nasal nitric oxide (nNO) owing to ostial occlusion and disruption of gas exchange with the nasal cavity. On the other hand, patients with CRSsNP have lower nNO than control patients but significantly higher than those observed in CRSwNP [127].

Patients with CRSwNP have decreased exhaled nasal nitric oxide (nNO) because of ostial occlusion and disruption of gas exchange [128]. Patients with CRSsNP have lower nNO than control patients but significantly higher than those observed in CRSwNP [127]. Atopy and allergic rhinitis further increase the nNO, complicating its role as a biomarker and minimizing its diagnostic utility [127].

The 22-Item Sinonasal Outcome Test (SNOT-22) is a validated CRS-specific health-related quality-of-life questionnaire that has become a mainstay in measuring outcomes in CRS, as it provides the highest quality disease-specific patient-reported outcome measure used in clinical practice compared to other validated CRS instruments [129]. As a result, SNOT-22 has been suggested to be incorporated into the clinical decision-making regarding the appropriateness of surgery, eligibility for a biologic, or both [1,130,131].

## 6. Chronic Rhinosinusitis Management

Since CRS is a chronic disease, the primary goal of management is to maintain clinically adequate disease control. Nasal irrigation with normal or hypertonic saline, nasal and systemic steroids, antibiotics, biologics, or surgical procedures can be applied according to the severity.

### 6.1. Nasal Irrigation

Nasal irrigation with isotonic saline or Ringer’s lactate is effective in CRS patient management [1]. The benefits of nasal saline irrigations include mechanical removal of inflammatory cells, mucus and crusts, blood, antigens/allergens, pollutants, and biofilms, improving mucociliary clearance, and enhancing ciliary beat activity [132]. High-volume irrigations seem more effective than low-volume saline sprays and nebulizers [133]. Although overall, saline irrigation appears to help treat CRS, there is contradictory evidence regarding the superiority of either hypertonic or isotonic solution for nasal douching on symptoms and mucociliary clearance [134,135].

### 6.2. Nasal Steroids

Nasal steroids suppress CRS inflammation by inhibiting proinflammatory transcription factors and mediators released from basophils and MCs [136]. They also reduce vascular permeability, inhibiting glycoprotein release from submucosal glands, and thinning mucus secretions [137]. The benefits of CRS therapy have the strongest level of evidence [138,139,140]. Penetration of nasal sprays beyond the nasal cavities into the paranasal sinuses has been shown to be limited. Data favor corticosteroid drops and corticosteroids delivered by nasal irrigation over corticosteroids sprayed intranasally [141,142,143]. Long-term administration of nasal steroids to the respiratory mucosa, evaluated by a systematic review (studies duration ranging from 5 days to 5.5 years), showed no evidence of damage to the nasal mucosa [144].

### 6.3. Systemic Steroids

Short courses of systemic corticosteroids for 1–3 weeks are associated with improved symptoms, quality of life, and reduced size of NP [145,146,147]. The effect on nasal polyp score remains significant up to three months after the start of treatment [1].

Short-term courses of systemic corticosteroids are generally safe, and according to the EPOS2020 steering group, one to two courses of systemic corticosteroids per year can be a useful addition to nasal corticosteroid treatment in patients with partially or uncontrolled disease [1]. However, the potential long-term side effects should always be taken into account [148]. Until now, there have been no randomized clinical trials for the usage of oral corticosteroids in CRSsNP.

### 6.4. Systemic Antibiotics

Systemic antibiotics are used in chronic rhinosinusitis to eliminate infection and inflammation, normalize the rheology and cohesivity of nasal mucus, alter bacterial biofilm formation, and reverse ostial occlusion [149]. Several systemic antibiotics, including penicillins, cephalosporins, quinolones, tetracyclines, and macrolides, have been studied for CRS treatment. A short course of doxycycline (200 mg once, then 100 mg daily for 20 days) for NP was associated with an improved polyp score 12 weeks after discontinuing treatment compared with placebo (*p* = 0.015) [150]. Macrolides can improve cilia function, inhibit the NF-ΚB pathway, and block binding to the TGF-β receptor apart from their direct antibacterial effects [137]. These immunomodulatory properties of macrolides are significant in CRSsNP treatment. Extended courses of low-dose macrolide antibiotics have been evaluated as chronic sinusitis therapy [151,152,153]. Based on these studies, macrolides demonstrate benefits in selected CRSsNP patients. Currently, no definitive biomarkers or prognostic factors exist for macrolide treatment selection in CRSsNP. The only RCT that included patients with NP showed no difference between macrolide therapy (azithromycin) and placebo [152]. Concerns have to be raised regarding gastrointestinal side effects [154], hepatotoxicity and ototoxicity [155], cardiac comorbidities [156], and the development of antibiotic resistance [157].

Despite the broad use of antibiotic therapy, acute bacterial rhinosinusitis may occur on top of CRS. These acute infections comprise the main cause of complications, which are typically classified as orbital (approximately 60–80%), intracranial (approximately 15–20%), and rarely osseous (approximately 5%) [158]. CRS inflammation per se has not been implicated in these types of complications but is a predisposing factor for acute rhinosinusitis complications, especially in adults [159].

### 6.5. Biologics

Biologic agents targeting T2 components, such as interleukins 4, 5, and 13, and IgE, offer new treatment approaches to manage severe refractory CRSwNP with or without comorbidities (e.g., asthma, atopic eczema, allergic rhinitis). There are currently three biologics registered in the EU and US as an add-on treatment of severe CRSwNP: omalizumab (anti-IgE) [160], mepolizumab (anti-IL5) [161], and dupilumab (anti-IL4/13) [162]. In all studies, the effect of the biologics was compared to a placebo added to continuous treatment with intranasal corticosteroids throughout the whole study period. The main primary endpoints (reduction in NP score and nasal congestion score) were met. Βiologics are best reserved for refractory cases and can be used in postoperative settings in CRSwNP patients. Preoperative consideration for those subtypes of CRSwNP with known refractory disease is debated [1,130].

In two recent phase 3 studies (SINUS-24 and SINUS-52), dupilumab, a human monoclonal antibody that inhibits IL-4 and IL-13 signaling, reduced the nasal polyp size and the severity of the sinonasal symptoms and improved olfactory dysfunction and quality of life in adult patients with severe CRSwNP [162].

Similarly, mepolizumab, a humanized monoclonal antibody that selectively inactivates IL-5, in the phase 3 study SYNAPSE, showed a significant improvement in nasal polyp size, nasal obstruction, and sinonasal symptoms, including sense of smell and quality of life in patients with CRSwNP under continuous medical treatment and previous nasal surgery [161].

Lastly, omalizumab, a humanized monoclonal antibody directed against free circulating IgE preventing its binding with the high-affinity IgE receptor FcεRI, in two phase 3 clinical trials (POLYP 1 and POLYP 2), significantly improved endoscopic, clinical, and patient-reported symptoms in adult patients with bilateral nasal polyps with weight and serum IgE levels permitting omalizumab dosing [160].

Although all the biologics mentioned above are indicated in patients with severe allergic or non-allergic asthma, improvement in nasal polyps was evident, independently of asthma or other allergic comorbidities. Furthermore, although all three significantly improved sinonasal symptoms, polyp size, and health-related quality of life, there was still a large number of non-responders, indirectly suggesting other underlying processes that are not dependent on IL-4-, IL-5-, IL-13-, and IgE-related pathomechanisms.

### 6.6. Targeting Janus Kinases

The Janus kinases (JAK) are a group of molecules composed of JAK1, JAK2, JAK3, and tyrosine kinase 2 (TYK2), which are key components within the JAK–signal transducers and activators of the transcription pathway, where cytokine receptor signaling takes place. JAK/STAT-mediated downstream events vary and include hematopoiesis, immune fitness, tissue repair, inflammation, apoptosis, and adipogenesis. More than 50 cytokines and growth factors have been identified in the JAK/STAT signaling pathway, such as hormones, interferons (IFN), and interleukins. This pathway is involved in the polarization of T helper cells and colony-stimulating factors [163,164] and could potentially be a target for future therapeutic options in CRS. It has been documented that phosphorylated STAT6 but not STAT1 and STAT3 are significantly increased in the sinonasal mucosa after allergen stimulation in a murine model of chronic eosinophilic airway inflammation [165,166], and STAT6 gene silencing ameliorated allergic rhinitis [167] and inhibited allergic airways inflammation [168,169]. In an interventional study of mice, topical tofacitinib administration was shown to be an effective treatment for eosinophilic CRSwNP by inhibiting phosphorylation, especially of STAT6, and decreasing the levels of eosinophil cationic protein and eotaxins [170].

### 6.7. Surgical Procedures

The minimally invasive sinus technique (MIST) has been introduced as a conservative approach involving simple ventilation of the lower sinuses. It should be applied only in selected cases with less aggressive endotypes of CRS. A recent study composed a combined model and predicted polyp recurrence after standard endoscopic sinus surgery, quantifying the prognostic value of eosinophil cationic protein, IL-5, pre-endoscopic sinus surgery-modified Lund–MacKay score, asthma, and anti-dsDNA IgG [171].

However, the weight of evidence for extended approaches lies in revision cases and not for primary surgery, especially concerning the frontal sinus, where a more conservative approach is usually deemed appropriate. Patients with aspirin-exacerbated respiratory disease (AERD) or non-steroidal anti-inflammatory drugs (NSAIDs)-exacerbated respiratory disease comprise an exception with failure rates after classic endoscopic sinus surgery as high as 90%. At least in these cases, extended approaches such as complete sphenoethmoidectomy and Draf III frontal sinus procedure may be justified [172]. The concept of the full resection of mucosa (reboot technique) in the most severe forms of CRS has been supported by some retrospective data but should be considered for patients with severe, uncontrolled CRSwNP when previous endoscopic sinus surgery efforts have failed [173].

## 7. Conclusions

Chronic rhinosinusitis with or without nasal polyps is a prevalent and heterogeneous disorder existing as a spectrum of clinical conditions with complex underlying pathomechanisms. CRS comprises a broad syndrome characterized by multiple immunological features involving complex interactions between the genes, the microbiome, host- and microbiota-derived exosomes, the epithelial barrier, and environmental and micromilieu exposures. The main pathophysiological feature is an epithelial barrier disruption, accompanied by microbiome alterations and unpredictable and multifactorial immunologic overreactions. Extrinsic pathogens and irritants interact with multiple epithelial receptors, which show distinct expression patterns, activate numerous signaling pathways, and lead to diverse antipathogen responses. CRSsNP is mainly characterized by fibrosis and mild inflammation and is often associated with Th1 or Th17 immunological profiles. CRSwNP appears to be associated with moderate or severe type 2 (T2) or Th2 eosinophilic inflammation. The diagnosis is based on clinical, endoscopic, and imaging findings. Possible CRS biomarkers from the peripheral blood, nasal secretions, tissue biopsies, and nasally exhaled air are studied to subgroup different CRS endotypes. The primary goal of CRS management is to maintain clinical control by nasal douching with isotonic or hypertonic saline solutions, administration of nasal and systemic steroids, antibiotics, biologics, and small molecules such as JAK inhibitors, or, in persistent and more severe cases, appropriate surgical procedures. The biologics targeting type 2 inflammation have already changed the therapeutic expectations in patients with recalcitrant to intranasal corticosteroids sinonasal disease and are expected to change the invasive algorithms by either minimizing the need for surgical interventions or enhancing and extending their outcomes in favor of CRS patients’ quality of life.

## Data Availability

Not applicable.
